# Molecular analysis of mucopolysaccharidoses: identification and characterization of pathogenic mutations in Indian population

**DOI:** 10.1186/1755-8166-7-S1-P60

**Published:** 2014-01-21

**Authors:** Anusha Uttarilli, S Jamal Md Nurul Jain, Ashwin B Dalal, Prajnya Ranganath, Shubha R Phadke, Girisha Kumar, Sankar, SJ Patil, Madhulika Kabra, Sumita Danda

**Affiliations:** 1 Diagnostics Division, CDFD, Nampally, Hyderabad, India

## Background

Mucopolysaccharidosis (MPS) are a group of rare inherited metabolic disorders which are caused due to the deficiency of a specific lysosomal enzyme involved in the catabolism of glycosaminoglycans. These disorders show a wide clinical spectrum ranging from severe, intermediate and mild phenotypes. Most of them show overlapping clinical features such as corneal clouding, coarse facies, hepatosplenomegaly, skeletal dysplasia, short stature, dysostosis multiplex, joint stiffness, joint contractures, cardiovascular and respiratory difficulties. Certain MPS disorders also show impaired neurological functions leading to mental retardation. They are inherited as an autosomal recessive with exception of MPS II, which is X-linked recessive disorder. They exhibit clinical, genetic as well as molecular heterogeneity. To date more than 200 mutations in IDUA, ~ 500 in IDS, ~ 200 in GALNS and ~ 200 in ARSB have been reported worldwide. The mutation spectrum of MPS disorders in Indian population is not characterized and established yet. This study was done to establish mutation spectrum in the Indian population that can be useful for the design of cost-effective strategies towards the molecular diagnosis of MPS disorders.

## Materials and methods

We have carried out mutation analysis of a total of 90 different MPS disorder patients (MPS I, II, IV and VI) and identified a total of 64 different mutations comprising majorly missense, nonsense, small deletions and splice site mutations. The mutations were further characterized using mutation prediction software and protein structure analysis for pathogenicity prediction.

## Conclusion

The characterization of mutational spectrum of Indian patients is likely to be useful in provision of better carrier diagnosis and prenatal diagnosis for patients with MPS disorders and also aids in designing of newer therapeutics for efficient treatment. It also helps in establishment of genotype-phenotype correlation and provision of better genetic counselling to the families with MPS affected members.

